# Supplementary material for: Text-mining forma mentis networks reconstruct public perception of the STEM gender gap in social media
Source: PeerJ Comput Sci. 2020 Sep 14;6:e295. doi: 10.7717/peerj-cs.295 (PMC7924458; doi:10.7717/peerj-cs.295)
Supplement: Supplemental Information 1 — The code describe the necessary resources and the procedure for building textual forma mentis networks. The code requires IGraphM in Mathematica 11.3. [file peerj-cs-06-295-s001.rtf]

%CODE for BUILDING TEXTUAL FORMA MENTIS NETWORKS

%This code was developed in Mathematica 11.3 by Massimo Stella. If using it, please cite the main manuscript where the code is described:

Stella, M. (2020). Text-mining forma mentis networks reconstruct public perception of the STEM gender gap in social media. arXiv preprint arXiv:2003.08835.


%AUXILIARY FUNCTIONS

valn=Import[NotebookDirectory[]<>"RatingsAffection.csv"];
changes=#[[1]]->#[[3]]&/@Import[NotebookDirectory[]<>"Changes.txt","Table",CharacterEncoding->"UTF-8"]
Mix[a_,b_]:=Partition[Riffle[a,b],2]
Draw[l1_,l2_,p_]:=Select[l1,MemberQ[l2,#[[p]]]&]
GraphBuild[l_]:=Graph[Flatten[{#[[1]]#[[2]]}&/@l]]
GraphDebuild[g_]:=Mix[EdgeList[g][[All,1]],EdgeList[g][[All,2]]]
Sorting[l_,p_]:=Reverse[SortBy[l,#[[p]]&]]
Combine[l_]:=Union[Flatten[l]]
RevSort[l_]:=Reverse[Sort[l]]
LCC[g_]:=Subgraph[g,ConnectedComponents[g][[1]]]
<<IGraphM`
IGDocumentation[]

%The main function also requires neg2, pos2, neu2, which are lists of words that are considered negative, positive and neutral, respectively. Notice the dependency of the code on the IGraphM package for using IGraph in Mathematica.


%MAIN FUNCTION

%The main function takes a vector of strings oo as an input and then proceeds building forma mentis networks sentence by sentence in each string. A temporary output is produced, outlining exactly which sentence in which string is currently being processed by the programme.

%The core of the function is grp, which uses the function TextStructure in order to extract syntactic dependencies between words. Dependencies are then classified according to the valence of the words between them and added in the graph variable gmer. 

%For network visualisation, it is possible to load a vector of word substitutions (changes) in order to change the name of words in the resulting network visualisation og, which is given as an output of this function.

netwe={};
Do[
 stn=oo[[j]];
 stn1=StringReplace[#,{".."->"."}]&/@TextSentences[stn];
 stn1=Select[stn1,StringLength[#]>40&];
 graphcol={};
 graphpos={};
 graphneg={};
 Do[

grp=TextStructure[stn,"DependencyGraphs"];
reded=WordStem/@Select[ToLowerCase/@(StringReplace[#,Table[ToString[i]->"",{i,0,VertexCount[grp[[1]]]}]]&/@GraphDebuild[grp[[1]]]),MemberQ[articles,#[[1]]]==False||MemberQ[articles,#[[1]]]==False&];
  reg=GraphBuild[reded];
  ly0=Union[Flatten[reded]];
  wordsto=Complement[Intersection[ly0,Join[valsexpan[[All,1]],{"not","no","you","we","I","me","us","they","them"},context]],prepositions,verbsaux];
  couples=Flatten[Table[{wordsto[[i]],wordsto[[j]]},{i,1,Length[wordsto]},{j,i+1,Length[wordsto]}],1];
  paths=FindPath[reg,#[[1]],#[[2]]]&/@couples;
  paths={Select[Flatten[paths,1],Length[#]<=4&]};
  affpath=Select[Select[paths,FreeQ["no"]],FreeQ["not"]];
  negpath=Complement[paths,affpath];
  affsemlin=First[#]<->Last[#]&/@Union[Flatten[affpath,1]];
  negsemlin=First[#]<->Last[#]&/@Union[Flatten[negpath,1]];
  afntw=Graph[wordsto,#[[1]]<->#[[2]]&/@affsemlin];
  nentw=Graph[wordsto,#[[1]]<->#[[2]]&/@negsemlin];
  totntw=Graph[wordsto,#[[1]]<->#[[2]]&/@Join[affsemlin,negsemlin]];
  fmv=VertexList[totntw];
  graphpos=Append[graphpos,afntw];
  graphneg=Append[graphneg,nentw];
  graphcol=Append[graphcol,totntw];
  PrintTemporary["Completed sentence:"<>ToString[stn]],
  {stn,stn1}];
 gmer=SimpleGraph[Graph[Union[Flatten[VertexList/@graphcol]]/.changes,Union[Flatten[EdgeList/@graphcol]]/.changes]];
 gsyn=Subgraph[gs,VertexList[gmer]];
 color1=RGBColor[0.01,0.5,1];
 color2=RGBColor[0.95,0.1,0.05];
 panelLabel[lbl_]:=Framed[Style[lbl,FontFamily->"Arial Narrow",If[lbl=="not"||lbl=="no",{Red,35},If[MemberQ[neg2,lbl]==True,{18,color2},If[MemberQ[pos2,lbl]==True,{18,color1},{18,Black}]]]],FrameMargins->1,RoundingRadius->5,FrameStyle->Directive[Opacity[0.2],GrayLevel[0.8]],Background->Directive[GrayLevel[0.95],Opacity[1]]];
 e1=EdgeList[gmer];
 negneg=Select[e1,MemberQ[neg2,#[[1]]]&&MemberQ[neg2,#[[2]]]&];
 pospos=Select[e1,MemberQ[pos2,#[[1]]]&&MemberQ[pos2,#[[2]]]&];
 neuneu=Select[e1,MemberQ[neu2,#[[1]]]||MemberQ[neu2,#[[2]]]&];
 negpos=Select[e1,MemberQ[neg2,#[[1]]]&&MemberQ[pos2,#[[2]]]||MemberQ[neg2,#[[2]]]&&MemberQ[pos2,#[[1]]]&];
 og=HighlightGraph[Graph[SimpleGraph[GraphBuild[Join[GraphDebuild[LCC[SimpleGraph[gmer]]],GraphDebuild[gsyn]]]],GraphLayout->{"EdgeLayout"->"HierarchicalEdgeBundling"},VertexLabels->Placed["Name",Center,panelLabel],VertexSize->0,EdgeStyle->Directive[Thickness[0.005],Opacity[0.5],Gray]],{Style[neuneu,Gray,Opacity[0.5],Thickness[0.01]],Style[negneg,color2,Opacity[0.8],Thickness[0.018]],Style[pospos,color1,Opacity[0.8],Thickness[0.018]],Style[negpos,Purple,Opacity[0.8],Thickness[0.018]],Style[EdgeList[gsyn],Green,Opacity[0.8],Thickness[0.018]]},ImageSize->600];
 netwe=Append[netwe,EdgeList[og]];
 PrintTemporary["Completed document number"<>ToString[j]],
 {j,{1}}]
